# Supplementary material for: Bulk delivery of a preassembled apical surface initiates epithelial lumen formation
Source: Nat Commun. 2026 Aug 7;17:9520. doi: 10.1038/s41467-026-75503-w (PMC13547361; doi:10.1038/s41467-026-75503-w)
Supplement: Supplementary file 2 [file 41467_2026_75503_MOESM2_ESM.pdf]

Title: Supplementary Data 1

Description: Mass spectrometry data of the 278 proteins identified by time-resolved Pals1-APEX2 proximity labelling, related to Figure 3.

Title: Supplementary Movie 1

Description: FIB-SEM of an intracellular VAC, related to Figure 6A

Title: Supplementary Movie 2

Description: 3D segmentation of microvilli in an intracellular VAC, related to Figure 6A

Title: Supplementary Movie 3

Description: FIB-SEM of a microvilli-containing membrane pit at the ECM-facing surface, related to Figure 6B

Title: Supplementary Movie 4

Description: 3D segmentation of peripheral ER and protrusions on the ECM-facing surface, related to Figure 6C
