# Supplementary material for: Bulk delivery of a preassembled apical surface initiates epithelial lumen formation
Source: Nat Commun. 2026 Aug 7;17:9520. doi: 10.1038/s41467-026-75503-w (PMC13547361; doi:10.1038/s41467-026-75503-w)
Supplement: Supplementary file 3 [file 41467_2026_75503_MOESM8_ESM.pdf]

nature portfolio

Corresponding author(s): Alexander Ludwig, Gunnar Dittmar

Last updated by author(s): 10.06.2026

Reporting Summary

Nature Portfolio wishes to improve the reproducibility of the work that we publish. This form provides structure for consistency and transparency in reporting. For further information on Nature Portfolio policies, see our Editorial Policies and the Editorial Policy Checklist.

Statistics

For all statistical analyses, confirm that the following items are present in the figure legend, table legend, main text, or Methods section.

n/a	Confirmed	
☐	☒	The exact sample size (n) for each experimental group/condition, given as a discrete number and unit of measurement
☐	☒	A statement on whether measurements were taken from distinct samples or whether the same sample was measured repeatedly
☐	☒	The statistical test(s) used AND whether they are one- or two-sided<br>*Only common tests should be described solely by name; describe more complex techniques in the Methods section.*
☒	☐	A description of all covariates tested
☐	☒	A description of any assumptions or corrections, such as tests of normality and adjustment for multiple comparisons
☐	☒	A full description of the statistical parameters including central tendency (e.g. means) or other basic estimates (e.g. regression coefficient) AND variation (e.g. standard deviation) or associated estimates of uncertainty (e.g. confidence intervals)
☐	☒	For null hypothesis testing, the test statistic (e.g. F, t, r) with confidence intervals, effect sizes, degrees of freedom and P value noted<br>*Give P values as exact values whenever suitable.*
☒	☐	For Bayesian analysis, information on the choice of priors and Markov chain Monte Carlo settings
☒	☐	For hierarchical and complex designs, identification of the appropriate level for tests and full reporting of outcomes
☒	☐	Estimates of effect sizes (e.g. Cohen's d, Pearson's r), indicating how they were calculated

Our web collection on statistics for biologists contains articles on many of the points above.

Software and code

Policy information about availability of computer code

Data collection	AiryScan imaging was performed on a ZEISS 980 LSM with Airyscan 2 operated in super-resolution mode using ZEISS Zen Software (v3.11). Confocal micrographs were acquired on a Corrsight Spinning Disk Microscope (Corrsight, ThermoFisher) using LA software (v2.7.0.16). TEM and CLEM data was acquired on a Tecnai T12 TEM using TIA software. FIB-SEM was performed on a Hitachi NX9000 FIB-SEM (by Hitachi Company). SEM was performed on a Quanta 650FEG (FEI). Mass spectrometry was performed on a Q-Exactive Plus mass spectrometer (Thermo Fisher Scientific).
Data analysis	Imaging data was processed and analysed with ImageJ (v2.16), ZEISS Zen Software (v3.11), and Adobe Photoshop (25.0 release). CellPose (https://github.com/mouseland/cellpose) was used for segmentation and quantification of apical actin levels in PatJ KO cells (Fig S12H). FIB-SEM data was segmented with Blender (v4.3.4). Fiji/ImageJ macros used to segment FIB-SEM data are available through Github: [https://github.com/Microscopy-Cluster-NUS/Microvilli_ER_vEM], target URL: [https://doi.org/10.5281/zenodo.20550192]. SEM data (Fig 7F and Fig S13) and Pals1 junctional levels in PatJ KO rescue lines (Fig S14D) were analysed using an automated pipeline in Phylo (https://biomni.phylo.bio). Plots and statistics were performed in Graphpad PRISM (v9.4.1) unless stated otherwise in the Methods.<br><br>Mass spectrometry data was analysed using MaxQuant (v1.6.1.0), R (v4.1.2), and ProTIGY, Broad Institute (https://github.com/broadinstitute/protigy). Data were searched against Canis Lupus Uniprot database (ID 9615). Enrichment analysis was carried out using the Enrichr analysis tool (https://maayanlab.cloud/Enrichr/). Protein interactions were retrieved from the STRING (v11.5) and BioGRID databases (v4.3.195). Protein Networks were drawn in Cytoscape (v3.10.3). Further data analysis and visualization were performed in R (v4.1.2). Data clustering and heatmap generation were carried out using the ComplexHeatmap package in R.

For manuscripts utilizing custom algorithms or software that are central to the research but not yet described in published literature, software must be made available to editors and reviewers. We strongly encourage code deposition in a community repository (e.g. GitHub). See the Nature Portfolio guidelines for submitting code & software for further information.

Data

Policy information about availability of data

All manuscripts must include a data availability statement. This statement should provide the following information, where applicable:

- Accession codes, unique identifiers, or web links for publicly available datasets
- A description of any restrictions on data availability
- For clinical datasets or third party data, please ensure that the statement adheres to our policy

All primary data supporting this manuscript will be publicly accessible through the NTU Data Repository (DR-NTU) upon publication [https://doi.org/10.21979/N9/VDWFBU]. The source data underlying Figures 1D, 1J, 1K, 4D, 4F, 4K, 6D, 6G, 7A, 7C, 7F, 8B, 8G, 9B, S2G, S12F, S12H, S13D, S13F, and S14D are provided as Source Data files. The mass spectrometry data generated in this study are provided in Supplementary File S1 and has been deposited in the PRIDE depository under accession code PXD061234, link [https://www.ebi.ac.uk/pride/archive/projects/PXD061234].

Research involving human participants, their data, or biological material

Policy information about studies with human participants or human data. See also policy information about sex, gender (identity/presentation), and sexual orientation and race, ethnicity and racism.

Reporting on sex and gender	NA
Reporting on race, ethnicity, or other socially relevant groupings	NA
Population characteristics	NA
Recruitment	NA
Ethics oversight	NA

Note that full information on the approval of the study protocol must also be provided in the manuscript.

Field-specific reporting

Please select the one below that is the best fit for your research. If you are not sure, read the appropriate sections before making your selection.

[x] Life sciences [ ] Behavioural & social sciences [ ] Ecological, evolutionary & environmental sciences

For a reference copy of the document with all sections, see nature.com/documents/nr-reporting-summary-flat.pdf

Life sciences study design

All studies must disclose on these points even when the disclosure is negative.

Sample size	No statistical method was used to predetermine sample size. Sample sizes varied across experiments, exact 'n' values are indicated in the figure legends and in the corresponding Source Data. Proteomics experiments sampled over 1 Mio cells per time point and technical replicate, which was sufficient to reproduce protein enrichment values between repeats. Light microscopy experiments were performed on large field of views to sample over 20 cells per experiment, which was sufficient to obtain reproducible mean values between experimental repeats. TEM, CLEM and SEM analyses was performed on large field of views, sampling more than 100 cells in each case.
Data exclusions	No data were excluded from the analyses unless stated otherwise. For mass spectrometry, proteins tagged as “Potential contaminants”, “Reverse”, and “Only identified by site” were removed from the analysis. Data was filtered to include only proteins with a minimum of two replicate identifications at each time point across the time course dataset. Proteins which were found to be significantly enriched with a p-value less than 0.05 and a log2 fold change greater than 1.0 as compared to both controls were considered for further analysis. For simplicity, the interactome network shown in Figure 3 shows only 200 proteins out of the 278 proteins identified.
Replication	TEM, SEM, CLEM, and FIB-SEM data are each derived from one biological replicate. All light microscopy and lumen phenotype data are derived from at least three independent biological replicates. Western blots shown in Figure 7A were performed three times. For all experiments replication attempts were successful.
Randomization	The experiments were not randomized. For LM and EM imaging analysis, areas of the monolayer were randomly imaged.
Blinding	LM experiments: The Investigators were not blinded to allocation during experiments and outcome assessment. SEM and FIB-SEM data was acquired by researchers who were not familiar with the sample.

Reporting for specific materials, systems and methods

We require information from authors about some types of materials, experimental systems and methods used in many studies. Here, indicate whether each material, system or method listed is relevant to your study. If you are not sure if a list item applies to your research, read the appropriate section before selecting a response.

Materials & experimental systems

n/a	Involved in the study
☐	☒ Antibodies
☐	☒ Eukaryotic cell lines
☒	☐ Palaeontology and archaeology
☒	☐ Animals and other organisms
☒	☐ Clinical data
☒	☐ Dual use research of concern
☒	☐ Plants

Methods

n/a	Involved in the study
☒	☐ ChIP-seq
☒	☐ Flow cytometry
☒	☐ MRI-based neuroimaging

Antibodies

Antibodies used

All antibodies used in this study including the supplier, catalogue and RRID numbers, are listed in Supplementary Table S1, which is included in the Supplemental Informations. Antibodies were used as follows:
Mouse monoclonal anti-Pals1 (clone G-5, Santa Cruz, sc-365411): IF: 1:150; WB: 1:2000
Rabbit polyclonal anti-PatJ (LSBio, LS-C410011): IF: 1:200; WB: 1:2000
Rabbit polyclonal anti-L27 (PatJ) (in house): WB: 1:1000
Mouse monoclonal anti-Ezrin (clone 3C12, Invitrogen, MA5-13862): IF: 1:200
Mouse monoclonal anti-Podocalyxin (DSHB, 3F2/D8): IF: 1:300
Mouse monoclonal anti-ZO1 (clone 1A12, ThermoFisher, Cat# 33-9100): IF: 1:200, WB: 1:2000
Rat polyclonal anti-ZO1 (in house): IF: 1:25
Rabbit polyclonal anti-Par3 (Merck, Cat# 07-330): IF: 1:500, WB: 1:5000
Rabbit polyclonal anti-CGN (clone C532, in house): IF: 1:2000
Mouse monoclonal anti-GAPDH (Invitrogen, Cat# sc-47724): WB: 1:5000

Validation

The specificities of anti-PatJ (LSBio, LS-C410011) and anti-Pals1 antibodies (clone G-5, Santa Cruz, sc-365411) were validated by Western blotting and immunofluorescence using PatJ and Pals1 KO MDCK cells (see Figures 7A, 7B, 7C, S12E and Groh et al., 2024, PMID: 38265145). The specificity of anti-ZO1 (clone 1A12, ThermoFisher, Cat# 33-9100) and anti-Par3 antibodies (Merck, Cat#07-330) were demonstrated previously using ZO1 KO MDCK cells (Tambrin et al., 2025, PMID 40198220) and Par3 siRNA (Tan et al., 2020, PMID 32531288). Rabbit anti-CGN (clone C532) and rat anti-ZO1 antibodies were gifted by Sandra Citi (Geneva) and had been validated before (Cardellini et al., 1996, PMID: 8875080). Rabbit anti-L27 (PatJ) antibody was gifted by Andre Le Bivic (Marseille) and was validated before (PMID: 11964389, PMID: 16129888) and in Figure S12D. Mouse anti-Ezrin (clone 3C12, Invitrogen, MA5-13862) and mouse anti-Podocalyxin (DSHB, 3F2/D8) antibodies were validated by the vendor.

Eukaryotic cell lines

Policy information about cell lines and Sex and Gender in Research

Cell line source(s)

MDCK-II cells were purchased from ECACC (Cat# 00062107). MDCK-II cells expressing endogenously tagged mNG-ZO1 were provided by Alf Honigmann (TU Dresden) and described previoulsy (Beutel et al., 2019, PMID 31675499). MDCK-II cells stably transfected with Pals1-APEX2-EGFP were described previously (Tan et al., 2020, PMID 32531288). MDCK-II Rab KO cells were purchased from RIKEN BRC and are described in Homma et al., 2019 (PMID: 31072826).

Authentication

Certificate of analysis of MDCK-II cells was provided by the vendor. All MDCK CRISPR KO clones were validated by WB, IF, and PCR/DNAsequencing of the guideRNA target locus.

Mycoplasma contamination

All cells and cell lines were Mycoplasma-negative, based on regular (every 2-3 months) tests performed with PCR-based mycoplasma detection kits

Commonly misidentified lines
(See ICLAC register)

No commonly misidentified lines were used

Plants

Seed stocks

Report on the source of all seed stocks or other plant material used. If applicable, state the seed stock centre and catalogue number. If plant specimens were collected from the field, describe the collection location, date and sampling procedures.

Novel plant genotypes

Describe the methods by which all novel plant genotypes were produced. This includes those generated by transgenic approaches, gene editing, chemical/radiation-based mutagenesis and hybridization. For transgenic lines, describe the transformation method, the number of independent lines analyzed and the generation upon which experiments were performed. For gene-edited lines, describe the editor used, the endogenous sequence targeted for editing, the targeting guide RNA sequence (if applicable) and how the editor was applied.

Authentication

Describe any authentication procedures for each seed stock used or novel genotype generated. Describe any experiments used to assess the effect of a mutation and, where applicable, how potential secondary effects (e.g. second site T-DNA insertions, mosiacism, off-target gene editing) were examined.
