# Supplementary material for: Bulk delivery of a preassembled apical surface initiates epithelial lumen formation
Source: Nat Commun. 2026 Aug 7;17:9520. doi: 10.1038/s41467-026-75503-w (PMC13547361; doi:10.1038/s41467-026-75503-w)
Supplement: Supplementary file 4 [file 41467_2026_75503_MOESM9_ESM.pdf]

nature portfolio

Peer Review File

Bulk delivery of a preassembled apical surface initiates epithelial lumen formation

Corresponding Author: Professor Alexander Ludwig

This file contains all reviewer reports in order by version, followed by all author rebuttals in order by version.

Version 0:

Reviewer comments:

Reviewer #1

(Remarks to the Author)
Dear Authors,

I am very impressed with the high-level microscopy and the elegant use of super-resolution and time-resolved proximity proteomics in this study. However, I have several substantial concerns that should be addressed to enhance both the rigor and reproducibility of the findings.

1. Lack of Quantification Across Figures:
There is a notable absence of quantitative analysis in the imaging data. For example, confocal microscopy results should include colocalization metrics—such as Manders' coefficient or Pearson's correlation coefficient to rigorously define spatial relationships between proteins. Including these analyses will strengthen interpretations and ensure clarity in the spatial dynamics of VACs and apical markers.

https://pubmed.ncbi.nlm.nih.gov/34616042/
https://pubmed.ncbi.nlm.nih.gov/34616045/

2. Use of FIB-SEM Data:
While the FIB-SEM imaging is visually compelling, the current presentation is limited to representative sectional views. To fully harness the power of FIB-SEM, 3D segmentation and volumetric quantification should be applied. There are existing tools and workflows (e.g., ilastik, Amira, and others cited in recent literature) that enable segmentation and quantification of organelles from FIB-SEM datasets. This would allow you to extract meaningful quantitative parameters from your structural data and more robustly support claims of VAC fusion and apical remodeling.
Example papers of this include the following:

https://www.nature.com/articles/s41467-024-48272-7

https://www.nature.com/articles/s41586-022-04488-5?fromPaywallRec=false

https://www.nature.com/articles/s41467-022-33678-y

https://www.nature.com/articles/s41467-022-30401-9

https://www.nature.com/articles/s41467-022-34445-9

https://www.nature.com/articles/s41467-018-07676-y

https://pubmed.ncbi.nlm.nih.gov/37960952/

3. Molecular Mechanisms of Fusion:
The manuscript speculates on the molecular machinery mediating VAC fusion, but direct experimental evidence is lacking. I

strongly recommend functional validation using siRNA or CRISPR/Cas9 knockouts of key candidate regulators such as Rab35, Rab11, Rab3, and SNARE components. This would provide mechanistic insight into vesicle fusion and validate the proposed model.

4. Role of Crumbs Complex vs. Pals1/PatJ Specificity:
While Pals1 and PatJ are known components of the Crumbs complex, their individual loss may exert effects independent of the full Crumbs complex. It would be important to test domain-specific deletion constructs to determine whether the observed phenotypes are mediated through direct Crumbs interaction or through other scaffolding functions. Rescue experiments using mutants of Pals1 or PatJ could clarify the functional domains required for VAC exocytosis and apical organization.

5. Validation of Proteomic Interactions:
To support the proteomic findings, I recommend validation of candidate protein-protein interactions via co-immunoprecipitation (co-IP) or proximity ligation assay (PLA). These techniques can confirm the proximity and interaction of VAC-associated proteins during apical domain initiation and provide confidence in the relevance of the proteomic network described.

6. Generalizability to Other Models:
Finally, to enhance the impact of this work, I suggest extending the analysis to another epithelial model system, such as Caco-2 intestinal epithelial cells. Alternatively, limited validation in organoid models could demonstrate physiological relevance. Even minimal data showing conservation of VAC dynamics or Pals1/PatJ function across systems would substantially strengthen the manuscript and broaden its relevance.

Reviewer #2

(Remarks to the Author)
One of the key features of epithelial cells is the assembly of the apical domain, with its unique suite of proteins, bounded at the margins by apical junctions. Defining the mechanisms by which the apical domain is assembled is a key goal. Here the authors combine a premier epithelial model, MDCK cells, with an exceptionally powerful set of imaging modalities, including superresolution microscopy, EM and CLEM, along with powerful time-resolved protemics, to analyze the process. While some of the observations here echo earlier work, the depth of the analysis and the combination of approaches provide a much clearer and thus novel set of insights. Their data strongly support the idea that in this cell type, in both 2D and 3D, the apical domain id defined by fusion of large vesicles preloaded with apical cargo and even possessing morphological features of the apical domain. They focus on defining the role the Crumbs complex proteins Pals1 and PatJ play, demonstrating important roles in the process. This work will be of broad interest to cell and developmental biologists. However, the breadth of the work means a few important points need to be explained more clearly, and a few remaining questions answered. We outline this below.

1. The authors carefully document the cargo in VACs after calcium switch. However, the initial endocytosis is described in less detail, simply stating “Pals started to coalesce at the cell membrane”? Where did this happen—at particular lateral membrane sites? Was this before or after adhesion was lost. Fig. 1A has many arrows, but there is little description of what they illustrate.

2. The Cartoon in Fig 2F is extremely helpful to understand the topological changes throughout the process. A similar cartoon at the very beginning in Fig 1, summarizing what’s already known (including AMIS-formation, PAP, VMZ) would make it much easier for non-experts to understand the system.

3. One thing that we found especially intriguing was the initial docking of VACs upon Calcium add-back. The authors suggest PALS1 is on microvilli in the VAC and ends in a ring around the apical patch when it is docked. However, this transition is unclear. Some closeups from imaging like that in Fig. 1E examining the stages in Fig. 1D would complement the remarkable EM images. This would also provide information about where ZO-1 localizes during this transition (Filling in the gap between the first two cartoon images in Fig. 2F) and make it clearer where on the lateral membrane VAC insertion occurs. It would also support the assertions in Fig 2 that the electron lucid regions are TJs. This would help explain the relatively late timing (i.e. after the claudins) of appearance of ZO-1 in the proximity proteomics.

4. Similar questions arose in the localization of PATJ/PALS1 and ZO-1 during initial assembly of the AMIS. Where to they localize between the stage sin Fig. 4C/D and 4E/F? When do they separate? We see a little of this for PATJ in Fig 4J/K, but what events lead to the focusing from the entire 2 cell interface to a region surrounding the exocytosed VAC? We mean the stage illustrated by the second panel in Fig 6. They even frame this question (p. 8) stating PATJ becomes enriched at the AMIS “prior to or concomitantly” to VAC delivery. This also is relevant to the treatments with Rho and other inhibitors—do they block docking, fusion of junctional assembly—they don’t need to fully answer this but clearer interpretation of their data would help.

5. There is a major topological difference between the 2D and the 3D setup and sometimes the authors explanations are too brief to allow the reader to full understand the intricacies.

In the 2D setup, many lateral membrane patches contacting different neighboring cells must somehow become one apical patch with TJ-contacts to all neighbors (topologically a ring) – that also somehow replaces the membrane there. This step is not trivial at all. It 1) either requires “fusion” of lateral lumens of different cell-cell interfaces at lateral 3-vertices. Subsequently, multi-cellular lumens could open towards the top and replace the membrane there. Alternatively, each

bicellular lateral lumen could fuse individually with the top "main lumen" generating multiple apical patches that must fuse with each other again to form one apical domain.
Do the authors have more information on how lateral membrane patches fuse to form the large apical "ring", that comprises TJ's contacting all neighbors? We are not recommending generating new data, but simply asking if more ifnromation on this is in the current data set. Regardless, some speculation about this would be helpful—this is the last step they depict in Fig 2F.

In contrast, in the 3D setup, there is no lateral lumen. The lumen between the two cells is the future apical side of the cells and does not change position. Instead, new cells that arise by cell division simply get a fraction of this lumen. Is this assumption correct, and if yes could the authors also add a cartoon depicting that, and make the (topological) differences between the 2D and the 3D setup clear in the text? And perhaps a little bit of speculation about whether additional VACs are added after the two cell stage.

6. The last Figure was a bit disconnected with the rest. This could be improved.

Minor points.
Fig 1—it would be helpful to make the panels larger, and perhaps add arrows? Also make it clearer at the start of the paper that you are beginning in 2D and using a calcium switch assay.

Fig 1D vesicles on both sides of the plasma membranes?

Fig 1 Captions. D in the last paragraph should be E.

In the text, the authors could add one or two sentences, introducing the calcium switch system, how removing calcium perturbs polarity, and that reintroducing it causes polarity to be re-established allowing to study lumen formation. This will make it easier to understand for non-experts.

Fig 4b is already the merge.

Add the merge in Fig 4C,D.

The interesting membrane staining used in Fig. 6B-E should be briefly described in the main text.

Reviewer #3

(Remarks to the Author)
I co-reviewed this manuscript with one of the reviewers who provided the listed reports. This is part of the Nature Communications initiative to facilitate training in peer review and to provide appropriate recognition for Early Career Researchers who co-review manuscripts.

Reviewer #4

(Remarks to the Author)
The study by Martin et al investigates the processes involved in apical lumen initiation in MDCK cells. Previous studies have yielded quite extensive insight into the molecular players and have suggested models for this process. Briefly, in MDCK cells the cytokinetic remnant (midbody) specifies a site at which the future apical membrane is formed (the apical membrane initation site, or AMIS). Future apical membrane proteins like Ezrin and podocalyxin are then endocytosed from the outer ECM-facing cortex, and transcytosed to the AMIS. This transcytosis was thought to be via standard vesicular transport involving Rab GTPases.

The authors here investigate the different steps of early apical domain formation in MDCK cells using a combination of various ultrastructural approaches and proteomics. They focus their studies on the Crumbs complex (Crumbs–Pals1–Patj). They first use a combination of light microscopy and TEM to investigate the localization of Pals1 at various stages of MDCK polarization. They do this first after a calcium switch in confluent MDCK monolayer, and later in the MDCK 3D cyst culture model. Unexpectedly, they find that Pals1 trafficks to the apical initiation site via large vacuolar apical compartments (VACs) rather than via endocytotic vesicles as previously postulated. Upon fusion of these VACs, Pals1 rapidly accumulates at nascent junction sites. The TEM studies are further elaborated on with FIB-SEM and CLEM microscopy approaches.

To move towards mechanistic insights, the ultrastructural imaging is complemented with APEX2 proximity ligation (with Pals1 as bait) at various time points after calcium switch. This analysis identifies distinct protein profiles at different times, that are consistent with the different steps of trafficking, fusion, and polarity establishment.

Next, the authors investigate the role of Pals1 and Patj in VAC trafficking. As previously reported, loss of either protein results in a multi-lumen phenotype. Imaging at the 2 cell stage indicates this is due to early defects, as knockout cells already show multiple large vacuoles in the doublet stage. The authors furthermore show that inhibiting Rho/ROCK signaling causes similar defects, leading them to postulate that Patj mediates VAC exocytosis through Rho/Rock signaling and actin organization.

All of the imaging and proteomics together convincingly show that VACs are present in MDCK cells in 2D and 3D MDCK cultures, and fuse at cell-cell contacts/AMIS. Timing wise, fusion and junction formation appear to happen close together in time or coincidentally. Together, the data indicate that our current thinking of MDCK polarization, and specifically the transcytosis steps, needs to be refined and incorporate that VACs are a major mode of transport of apical proteins. The data also fit with other papers on pre-assembled apical vesicle structures that are well discussed. The manuscript should therefore be of broad interest to others working on epithelial organization.

Regarding the imaging: I have a question related to the observation of microvilli. Are the authors sure that the structures in the VACs represent microvilli? I ask for two reasons. First, the appearance of these protrusions seems more irregular than I would expect. I do not see actin bundles, and in the supplementary videos the villi often look connected, almost like a network of connected tubules or stacks. Second, in in the proximity proteomics, microvillar proteins like EPS8L1, EPS8, BAIAP2, and BAIAP2L1 are detected late, in cluster 3, and not in clusters 1 and 2 during VAC trafficking. Does this not indicate that microvilli are formed only after fusion? After all, the bait protein Pals1 is present on the protrusions during the trafficking phases.

In addition, images in previous publications indeed often are consistent with VACs and as the authors suggest, may simply not have been recognized. Yet there are aspects of previous models of MDCK polarization and transcytosis of apical factors that need to be resolved between prior studies and this manuscript. In particular, work from Bryant and Mostov (2014) suggests that during transcytosis, Ezrin and PODXL do not colocalize. In this manuscript, both are suggested to be present in VACs but a double labeling is not shown. Can the authors resolve whether Ezrin and PODXL traffic together or not?

Regarding the conclusions related to mechanistic insights, I am less convinced by the experiments. The first major conclusion is that Pals1 and Patj loss impairs VAC exocytosis at the AMIS. This conclusion is based on the observation of 'large intracellular vacuoles' and 'ectopic or possibly intracellular lumens' in doublets of knockouts. This could equally well be due to problems with VAC formation or trafficking in the cell, not necessarily with the exocytosis step. Especially since I do not see the suggested accumulation of VACs at the AMIS, but they appear dispersed through the cell.

The second conclusion is that exocytosis defects may be due to defective Rho/RHOCK signaling. To support this, they show that inhibiting Rho or ROCK leads to similar defects as knockout of Pals1 or Patj (accumulation of VACs). In addition, in 2D cultures, knockdown of Patj leads to reduced apical F-actin levels. One issue is that the Rho inhibition results appear qualitatively different from the Patj knockout data (compare 7D with 7F): the Rho inhibition vesicles do appear to cluster at the AMIS. But more importantly, the fact that both treatments result in lumen initiation defects does not directly imply that they are functionally related. While Crumbs is indeed involved in Rho/ROCK signaling, the functions of Crumbs are complex and pleotropic. As an apical domain specifying factor and regulator of junctions and actin, the reduced apical actin levels can easily be explained by a lack of Pals1/Patj at the apical membrane domain.

The discussion part of the roles of Pals1 and Patj was also confusing to me. The model presented here is that Pals1 and Patj are at cell-cell contacts, and then VACs deliver apical cargo including Crb3. Assembly of the full Crumbs complex would only take place after VACs fuse at the AMIS. The authors reconcile their finding with previous data concluding that all three Crumbs components traffic via Rab11-positive carriers by stating that the current study is done using antibodies, and previous studies with GFP fusions. However, figures 1 and 2 show that Pals1 is present in VACs. Crb3 and Pals1 would therefore be together in the same compartment. It seems more likely to me that the full Crumbs complex is present in VACs. In addition, for Crb3 the authors rely for their conclusion on the same studies they criticize for using GFP fusions. If the Pals1 and Patj data are not trustworthy, why would the Crb3 data be?

The authors also suggest that Pals1 may act as the VAC cargo receptor by binding to Crb3. Do they mean the receptor that mediates VAC fusion at the AMIS or a receptor that mediates loading of Crb3 into VACs? The first seems unlikely since Crb3 and Pals1 are both in VACs. The second is possible but would have no bearing on the delivery of VACs.

Before publishing this work, it will be necessary to either perform additional work to strengthen the mechanistic insights, or to significantly tone down the mechanistic conclusions. In either case, the confusing contradiction in the discussion should be resolved. To strengthen the mechanistic insights, the authors should show that loss of Pals1 specifically affects exocytosis by performing time-lapse imaging of VACs and demonstrating that their trafficking to the AMIS is not affected. In addition, the authors should show that composition and structure of VACs is not affected by Pals1 loss. That the defects in Rho signaling and Crumbs complex loss are functionally related also needs to be substantiated. For example, can the Pals1 defects be restored by increasing Rho activity?

Version 1:

Reviewer comments:

Reviewer #1

(Remarks to the Author)
Dear Authors,

I want to commend each of you on the tremendous effort that has gone into this manuscript. It is an innovative and impactful study, and it is clear that a great deal of thought and care was invested in both the experimental design and analysis.

I especially appreciate the rigor of the quantitative analyses and the meaningful integration of advanced approaches such as FIB-SEM. The revisions demonstrate a strong commitment to addressing reviewer feedback and have significantly strengthened the manuscript.

Overall, this is a well-executed and high-quality study, and I commend the team for their outstanding work.

Reviewer #2

(Remarks to the Author)
In our initial review we noted that one of the key features of epithelial cells is the assembly of the apical domain, with its unique suite of
proteins, bounded at the margins by apical junctions. Defining the mechanisms by which the apical domain is assembled is a key goal. Here the authors combine a premier epithelial model, MDCK cells, with an exceptionally powerful set of imaging modalities, including superresolution microscopy, EM and CLEM, along with powerful time-resolved protemics, to analyze the process. Their data strongly support the idea that in this cell type, in both 2D and 3D, the apical domain id defined by fusion of large vesicles preloaded with
apical cargo and even possessing morphological features of the apical domain. They focus on defining the role the Crumbs complex proteins Pals1 and PatJ play, demonstrating important roles in the process. The authors have addressed all of our earlier concerns and this work will be of broad interest to cell and developmental biologists.

Reviewer #3

(Remarks to the Author)
I co-reviewed this manuscript with one of the reviewers who provided the listed reports. This is part of the Nature Communications initiative to facilitate training in peer review and to provide appropriate recognition for Early Career Researchers who co-review manuscripts.

Reviewer #4

(Remarks to the Author)
I thank the authors for their extensive clarifications and experiments in response to the point I raised. I have no further points.

Open Access This Peer Review File is licensed under a Creative Commons Attribution 4.0 International License, which permits use, sharing, adaptation, distribution and reproduction in any medium or format, as long as you give appropriate credit to the original author(s) and the source, provide a link to the Creative Commons license, and indicate if changes were made.

In cases where reviewers are anonymous, credit should be given to 'Anonymous Referee' and the source.
The images or other third party material in this Peer Review File are included in the article's Creative Commons license, unless indicated otherwise in a credit line to the material. If material is not included in the article's Creative Commons license and your intended use is not permitted by statutory regulation or exceeds the permitted use, you will need to obtain permission directly from the copyright holder.
To view a copy of this license, visit https://creativecommons.org/licenses/by/4.0/

Detailed response to the reviewers (in bold):

Reviewer #1 (Remarks to the Author):

Dear Authors,

I am very impressed with the high-level microscopy and the elegant use of super-resolution and time-resolved proximity proteomics in this study. However, I have several substantial concerns that should be addressed to enhance both the rigor and reproducibility of the findings.

1. Lack of Quantification Across Figures:
There is a notable absence of quantitative analysis in the imaging data. For example, confocal microscopy results should include colocalization metrics—such as Manders' coefficient or Pearson's correlation coefficient to rigorously define spatial relationships between proteins. Including these analyses will strengthen interpretations and ensure clarity in the spatial dynamics of VACs and apical markers.

Response: We agree with the reviewer that a thorough quantitative analysis of our imaging data was lacking. We have addressed this shortcoming systematically and comprehensively in the revised manuscript. We have quantified the colocalization between Pals1/PatJ (VMZ markers), PODXL/phalloidin (apical and VAC marker), and ZO-1/cingulin (TJ markers) in both the Ca-switch experiments and during 3D cyst development using Pearson's Correlation. We analysed endogenous proteins using specific antibodies and confirmed this in MDCK cell lines stably expressing Pals1-GFP and in MDCK CRISPR knockin cell lines expressing endogenously tagged mNeonGreen-ZO-1.

In the Ca-switch (2D) experiments, we confirm that endogenous Pals1, PatJ, and PODXL are localised to VACs in calcium depleted cells (Fig1 E-J and Fig S2). Once calcium is given back to the cells, VACs are delivered to and fuse at nascent cell contacts. At this stage, the colocalisation between Pals1/PatJ and PODXL/actin drops, and Pals1/PatJ "move" into the lateral membrane to form a junctional "ring" around the VAC fusion site that also contains the TJ markers ZO-1 and cingulin (CGN). Consistent with our serial section TEM data, these fusion sites then expand/grow along the membrane to form a lateral lumen surrounded by cell junctions. Importantly, our new colocalization analysis clearly shows that Pals1 and PatJ are spatially separated from ZO1 and CGN, with both VMZ markers localising closer to the PODXL-labeled lateral lumen (Fig 1H and 1I and Fig S2). This shows that Pals1 and PatJ become associated with TJ proteins as soon as cell junctions are assembled and the lateral lumen is formed, and that the two domains (VMZ and TJ) are spatially separated, even at this early stage of polarity development.

To analyse the localisation of Pals1, PatJ and ZO-1 in 3D cysts, we quantified their colocalisation at two stages - the inverted stage in which TJ and VMZ proteins are localised at the periphery of the cell-cell contact zone (facing the ECM), and the PAP stage, in which TJ and VMZ proteins surround the nascent apical luminal domain. Intriguingly, and in line with our data from the Ca switch experiments, we find that at both stages Pals1 and PatJ colocalise significantly more strongly with each other than with ZO-1 (Fig 4E-K and Fig S7). This shows that TJ and VMZ proteins form separate domains even before the apical lumen is formed.

Our new analysis also revealed that TJ and VMZ proteins switch positions during the AMIS-PAP transition (Fig 4E-J and Fig S7). In the "early inverted" stage, Pals1 and PatJ face outward, toward the ECM, whilst ZO-1 faces inward, toward the midbody (Fig 4E). In the "late inverted" stage, just before the AMIS starts to emerge, TJ and VMZ proteins spread along the cell-cell contact interface, and at this stage, ZO1 faces toward the midbody, whilst Pals1/PatJ face outward (Fig 4H). This suggests that the two domains "move across each other". At the AMIS stage (Fig 4I), TJ and VMZ proteins all cluster around the midbody and then switch orientation in the PAP stage (Fig 4J), with Pals1/PatJ facing the lumen and ZO-1 facing outward (toward the then established lateral membrane interface). This is – to our knowledge – the first visual demonstration of the "polarity switch" during lumen initiation.

Importantly, unlike in the Ca switch assay in which Pals1 and PatJ become internalised into VACs, <u>Pals1 and PatJ are not present in VACs in the 3D system (Fig 5A-D)</u>. To provide additional evidence that VACs are transport devices for apical cargo, we have analysed additional apical proteins (PODXL, ezrin, and Crb3-GFP) using antibody uptake assays and dual/triple immunostainings. These new data clearly show that PODXL, ezrin, and Crb3 are co-transported in VACs (Fig 6E-6H and Fig S11).

Altogether, this comprehensive quantitation substantiates that the VMZ and TJ proteins form separate cortical domains and that this separation is evident before cells are fully polarised. In addition, the data support our model that VACs are apical precursor organelles that are delivered to nascent cell-cell contacts, where they fuse to specify the apical luminal domain.

2. Use of FIB-SEM Data:
While the FIB-SEM imaging is visually compelling, the current presentation is limited to representative sectional views. To fully harness the power of FIB-SEM, 3D segmentation and volumetric quantification should be applied. There are existing tools and workflows (e.g., ilastik, Amira, and others cited in recent literature) that enable segmentation and quantification of organelles from FIB-SEM datasets. This would allow you to extract meaningful quantitative parameters from your structural data and more robustly support claims of VAC fusion and apical remodeling.

Response: We thank the reviewer for this suggestion. We have segmented parts of the FIB-SEM volume to visualise the 3D organisation of a) microvilli inside the VACs, b) membrane pits enriched in microvilli, and c) membrane protrusions on the ECM-facing plasma membrane surface. This new data is shown in Figure 6A-6D.

To confirm that the membrane protrusions we observe inside the VACs are indeed microvilli, we measured their diameter (width) and compared their dimensions to apical microvilli found in mature MDCK cell monolayers. Previous work has demonstrated that apical microvilli are ~100 nm in diameter, whilst filopodia-like protrusions are more heterogenous in size (100-300 nm in width) (Mooseker and Tilney 1975, PMC2111646; Morales et al., 2023, PMC10033394).

Our analysis shows that the membrane protrusions found inside VACs and inside the ECM-facing membrane pits are ~110 nm in diameter, exactly matching the dimensions of microvilli on the apical surface of fully mature MDCK cells grown on filter inserts and those reported by

others. By contrast, with an average diameter of ~140 nm (Fig 6D), the membrane protrusions found on the ECM-facing plasma membrane were significantly larger.

This provides strong evidence that the membrane protrusions inside VACs are indeed microvilli. We further conclude from this analysis that apical microvilli are present on the ECM facing PM in two-cell stage MDCK cysts. Through a mechanism that is currently unknown, these microvilli are somehow efficiently sequestered in micrometer-scale membrane pits, which are subsequently internalized to form VACs. All in all, the data is consistent with a model in which apical cortex containing microvilli is internalized from the ECM-facing PM and transported to the AMIS via VACs.

3. Molecular Mechanisms of Fusion:
The manuscript speculates on the molecular machinery mediating VAC fusion, but direct experimental evidence is lacking. I strongly recommend functional validation using siRNA or CRISPR/Cas9 knockouts of key candidate regulators such as Rab35, Rab11, Rab3, and SNARE components. This would provide mechanistic insight into vesicle fusion and validate the proposed model.

Response: We thank the reviewer for raising this point. To address the functions of apical Rabs in lumen formation, we analysed several MDCK Rab KO cells, which had previously been generated and characterised by the Fukuda lab (Homma et al., JCB 2020, PMID: 31072826). We analysed Rab11a/b double KOs, Rab3a/b/c/d quadruple KOs, Rab35, and Rab25 KOs. None of these Rab KO lines showed defects in apical lumen formation in the 3D cyst assay; most mature cysts (3-4 days in Matrigel) showed a single apical lumen. In addition, we did not observe any defects in PAP formation at the two-cell stage (24h). The data is summarized in Figures S15A and S15B and is briefly described in the discussion.

The lack of a lumen phenotype is largely in line with Homma et al, who analysed cyst morphogenesis in all Rab KO cell lines grown in collagen matrix (in which lumens are formed through apoptosis in the centre of a spheroid, and not through apical protein trafficking). None of the Rab KOs they analysed, except Rab11a/b dKOs, showed a lumen formation defect. Their and our data clearly suggest that there is significant functional redundancy in the Rab protein family. This redundancy seems to become evident in KO cells but not in cells in which Rabs are silenced through siRNAs or shRNAs, as there is ample evidence that downregulation of apical Rabs (e.g. Rab11 and Rab25) perturbs the trafficking of apical cargo and apical domain assembly in cells and tissues (see e.g. Bryant et al., 2010).

In addition, we addressed the function of the exocyst complex in VAC exocytosis and lumen morphogenesis, using the EXO70 inhibitor Endosidin-2. Cells treated with Endosidin-2 tended to establish cysts with more than one lumen and exhibited altered lumen shapes (see Fig S15C-S15G), in agreement with previous work showing that exocyst functions at the AMIS (see e.g. Bryant et al., 2010).

We hope the reviewer understands that dissecting the mechanisms of Rab redundancy and those underlying membrane fusion at the AMIS goes beyond the scope of the current manuscript.

4. Role of Crumbs Complex vs. Pals1/PatJ Specificity:
While Pals1 and PatJ are known components of the Crumbs complex, their individual loss may exert

effects independent of the full Crumbs complex. It would be important to test domain-specific deletion constructs to determine whether the observed phenotypes are mediated through direct Crumbs interaction or through other scaffolding functions. Rescue experiments using mutants of Pals1 or PatJ could clarify the functional domains required for VAC exocytosis and apical organization.

Response: To address this, we have performed rescue experiments in our PATJ KO cells using previously established mCherry tagged PatJ constructs (Pombo-Garcia et al., 2024; PMID: 39112699). Pombo-Garcia (2024) demonstrated that CRISPR/Cas9-mediated deletion of the L27 domain of PatJ (which is required for the interaction with Pals1) severely disrupts TJ formation in MDCK cells. The authors show that PatJ constructs containing the L27 and PDZ6 domains of PatJ are required and sufficient to rescue the TJ defect. In addition, a chimeric construct in which the L27 domain of PatJ was fused to ZO-1 rescued the TJ defect in their PATJ KO cells. It was concluded that PatJ is essential for TJ formation by establishing a physical link between the apical Crumbs complex (L27 mediated) and the ZO-1 belt (PDZ6 mediated).

We find that none of the PatJ constructs made by Pombo-Garcia (2024), including full-length PatJ, localise to apical cell junctions or rescue the multi-lumen phenotype in _our_ PATJ KO cells (Fig 9A+B and S14). The only construct that was efficiently targeted to apical junctions and fully rescued the multi-lumen defect was the chimeric PatJ-ZO1 construct (Fig 9A+B and S14). The chimera also restored AMIS assembly and VAC trafficking to the AMIS (Fig 9C). Moreover, the PatJ-ZO1 chimera, but not full-length PatJ, efficiently recruited endogenous Pals1 back to apical cell junctions (Fig S14C+D).

Why is PatJ not targeted to cell junctions in our PATJ KO cells? We propose that this is because junctional Pals1 and ZO-1 levels are both markedly reduced in PATJ KO cells (Fig 7A-C, Fig S12E+F). In the absence (or the presence of sub-stoichiometric amounts) of both the TJ anchor (ZO-1) and the apical anchor (Pals1), PatJ cannot be effectively retained at the membrane. Ectopic re-expression of the chimera overcomes this problem because the PatJ L27 domain is now anchored at the membrane via ZO-1, which likely promotes the L27/L27 domain interaction between PatJ/Pals1 at the apical-lateral interface.

We would further like to stress that there are important differences between the Pombo-Garcia PatJ L27 deletion cells and our PATJ KO cells. The L27 domain deletion introduced by Pombo-Garcia results in the expression of a PatJ protein encompassing all PDZ domains but lacking the L27 domain (which the authors show in the Supplemental Information of their paper). By contrast, our PATJ KO cells were generated with a guideRNA targeting exon 11, which causes a frame shift and a premature stop codon just downstream of the PDZ3 domain (see Fig S12). We show (using anti-PDZ4 and anti-L27 domain antibodies) that this leads to the complete loss of PatJ expression (Fig 7A and Fig S12D). Hence, our PATJ KO cells are a true "loss-of-function model", whereas deletion of the L27 domain and expression of a truncated PatJ protein may cause a "dominant-negative effect" and therefore interfere with TJ formation in a manner distinct from our PATJ KO cells. Indeed, compared to L27 deletion cells, our PATJ KO cells show a relatively mild TJ formation defect in 2D cultures (Fig S12 and S14). These differences and differences in PatJ expression levels in rescue lines may explain why PatJ constructs do not rescue the lumen formation defects in our PATJ KO cells.

Notwithstanding these technical discrepancies, our data and the data by Pombo-Garcia are clearly complementary and together demonstrate that PatJ is critical for TJ formation and

lumen morphogenesis. Importantly, it was previously shown that ZO1/2 double KO MDCK cells, which are severely compromised in TJ belt formation and barrier function (e.g. Otani et al 2019, PMID: 31467165), still form a single lumen in Matrigel, albeit the lumen is deformed due to a hypercontractile junctional belt (e.g. Mukenhirn 2024, PMID: 39137775). This shows that a functional TJ belt is not absolutely required for apical lumen initiation, in turn indicating that the PAP formation defect we observe in our PATJ KO cells is not caused by defects in TJ assembly per se.

Instead, we propose that PatJ's primary function is to link the TJ to the apical cortex and to stabilize the apical-lateral border. In support of this, comprehensive quantification of our SEM data revealed that loss of PatJ markedly alters the architecture of the marginal zone (Fig 7, Fig S13). In addition, we show that TJ proteins are not recruited to or retained at the AMIS in the absence of PatJ, preventing VAC incorporation and apical lumen assembly (Fig 8). We conclude that the marginal zone mediates (or is the ultrastructural manifestation of) the physical linkage between the Crumbs complex and the TJ belt, and that this linkage is critical for apical lumen initiation (Fig 9D).

5. Validation of Proteomic Interactions:
To support the proteomic findings, I recommend validation of candidate protein-protein interactions via co-immunoprecipitation (co-IP) or proximity ligation assay (PLA). These techniques can confirm the proximity and interaction of VAC-associated proteins during apical domain initiation and provide confidence in the relevance of the proteomic network described.

Response: We have validated our proteomics time course in various ways.

1. **We show using light and electron microscopy that the apical Rab proteins Rab3, 11, 25, and 35, all of which were identified by proteomics, localise to VACs (Fig S6). In an attempt to understand whether these Rabs are important for VAC transport and lumen formation, we analysed MDCK Rab KO lines (see response to question 3 and Figure S15).**
2. **We have analysed and quantified the localisation and colocalization of VMZ, TJ and apical proteins in the calcium switch experiment using Pearson's correlation (see response to question 1). The new imaging data correlates very well with the proteomics data and with the ultrastructural changes we observe in serial section TEM.**

We have not moved beyond the above to explore protein-protein interactions of Pals1/PatJ because this had actually been done in our previous work (Tan et al., Current Biology 2020). The objective of the proteomics approach in the current manuscript was to resolve the molecular events that occur during polarity development, using Pals1 as a VMZ proxy and APEX2 labeling to effectively label proximal proteins. Hence, the time-resolved proteome of Pals1 generated with APEX2 should not be misinterpreted as a Pals1 "interactome". In fact, most proteins identified in this proteome are not directly or even indirectly associated with Pals1 but instead are enriched in its proximity at different time points of the assay.

In addition, in our experience, PLA is not be suitable to analyse the association of VMZ and TJ proteins, as these two domains are connected but spatially separated along the apical-lateral border (note that PLA efficiency decreases at distances > 20 nm).

We hope the reviewer understands that dissecting protein-protein interactions in this network goes beyond the scope of this manuscript.

6. Generalizability to Other Models:
Finally, to enhance the impact of this work, I suggest extending the analysis to another epithelial model system, such as Caco-2 intestinal epithelial cells. Alternatively, limited validation in organoid models could demonstrate physiological relevance. Even minimal data showing conservation of VAC dynamics or Pals1/PatJ function across systems would substantially strengthen the manuscript and broaden its relevance.

Response: To address this, we performed calcium switch experiments in Caco-2 cells. Upon depletion of calcium, ezrin antibodies and phalloidin stained intracellular vacuoles reminiscent of VACs (Fig S1F). This suggests that VAC formation is a general and widespread feature of epithelial cells.

As we outline in the discussion, large intracellular apical vacuoles reminiscent of VACs have been observed in various other cell types in culture (e.g. human ES cells; Taniguchi et al 2017) and in vivo (e.g. in the mouse axial mesoderm; Gredler et al 2023), and in epiblast/blastocyst models (e.g. Ryan et al 2019). A detailed analysis of the dynamics and structure-function relationship of VACs in physiological settings requires high-resolution 3D imaging (TEM or super-resolution) and appropriate ES cell or in vivo models, which will take time to establish. We hope this reviewer understands that such elaborate experiments are beyond the scope of the current manuscript.

Reviewer #2 (Remarks to the Author):

One of the key features of epithelial cells is the assembly of the apical domain, with its unique suite of proteins, bounded at the margins by apical junctions. Defining the mechanisms by which the apical domain is assembled is a key goal. Here the authors combine a premier epithelial model, MDCK cells, with an exceptionally powerful set of imaging modalities, including superresolution microscopy, EM and CLEM, along with powerful time-resolved protemics, to analyze the process. While some of the observations here echo earlier work, the depth of the analysis and the combination of approaches provide a much clearer and thus novel set of insights. Their data strongly support the idea that in this cell type, in both 2D and 3D, the apical domain id defined by fusion of large vesicles preloaded with apical cargo and even possessing morphological features of the apical domain. They focus on defining the role the Crumbs complex proteins Pals1 and PatJ play, demonstrating important roles in the process. This work will be of broad interest to cell and developmental biologists. However, the breadth of the work means a few important points need to be explained more clearly, and a few remaining questions answered. We outline this below.

1. The authors carefully document the cargo in VACs after calcium switch. However, the initial endocytosis is described in less detail, simply stating "Pals started to coalesce at the cell membrane"? Where did this happen—at particular lateral membrane sites? Was this before or after adhesion was lost. Fig. 1A has many arrows, but there is little description of what they illustrate.

Response: We have updated the panel and the labels and now describe the process in more detail in the results section (see Fig 1B). The purple arrowhead labels an existing VAC, the red arrowhead labels the newly formed VAC. The initial coalescence of Pals1 occurs way after the cells have lost polarity (after cell junctions are lost), and this coalescence happens at the apical (or now better called dorsal) pole of the cells. This is now illustrated in the x/z projections we have included in the panel.

We also repeated these experiments in the presence of PODXL antibody pre-labelled with a far-red fluorescent dye (equivalent to our antibody uptake experiments in 3D cultures), showing that PODXL and Pals1 are co-internalized into VACs (see Fig S1A). This supports our conclusion that VACs receive apical cargo through internalization from the plasma membrane.

2. The Cartoon in Fig 2F is extremely helpful to understand the topological changes throughout the process. A similar cartoon at the very beginning in Fig 1, summarizing what's already known (including AMIS-formation, PAP, VMZ) would make it much easier for non-experts to understand the system.

Response: We thank the reviewer for this suggestion. A cartoon illustrating the principle of the Ca switch model is now shown in Figure 1A. Questions regarding the VMZ and the delivery of VACs to the cortex are highlighted. We also show in Figure 4A a cartoon illustrating the currently accepted model of lumen formation in the 3D MDCK cysts.

3. One thing that we found especially intriguing was the initial docking of VACs upon Calcium add-back. The authors suggest PALS1 is on microvilli in the VAC and ends in a ring around the apical patch when it is docked. However, this transition is unclear. Some closeups from imaging like that in Fig. 1E examining the stages in Fig. 1D would complement the remarkable EM images. This would also provide information about where ZO-1 localizes during this transition (Filling in the gap between

the first two cartoon images in Fig. 2F) and make it clearer where on the lateral membrane VAC insertion occurs. It would also support the assertions in Fig 2 that the electron lucid regions are TJs. This would help explain the relatively late timing (i.e. after the claudins) of appearance of ZO-1 in the proximity proteomics.

Response: We thank the reviewer for raising this point and agree this analysis was rudimentary. To address what is happening at the VAC fusion state, we have imaged additional TJ proteins (ZO-1 and cingulin (CGN)) and carefully quantified the colocalization of these with Pals1/PatJ in the calcium switch assay (see also response to Reviewer 1). The new data is now presented in Figure 1E-1J (and Fig S2). We find that Pals1 and PatJ as well as ZO1 and CGN are all present in the junctional ring that surrounds the VAC fusion sites and the lateral lumen. Intriguingly, we see a clear spatial separation of the VMZ and TJ proteins, even at these early stages, with Pals1 and PatJ being closer to the VAC or lumen, as expected (depicted in the model in Fig 1J). This indicates that the VMZ proteins are separated from TJ proteins even before the apical junctional belt is fully formed. It also shows that apical lumen initiation in 2D and 3D follows a similar mechanism with regards to cell-cell junction assembly and delivery of apical cargo.

4. Similar questions arose in the localization of PATJ/PALS1 and ZO-1 during initial assembly of the AMIS. Where do they localize between the stages in Fig. 4C/D and 4E/F? When do they separate? We see a little of this for PATJ in Fig 4J/K, but what events lead to the focusing from the entire 2 cell interface to a region surrounding the exocytosed VAC? We mean the stage illustrated by the second panel in Fig 6. They even frame this question (p. 8) stating PATJ becomes enriched at the AMIS "prior to or concomitantly" to VAC delivery. This also is relevant to the treatments with Rho and other inhibitors—do they block docking, fusion or junctional assembly—they don't need to fully answer this but clearer interpretation of their data would help.

Response: We thank the reviewer for this comment. To address this, we have quantified the colocalization between VMZ and TJ markers at different stages of lumen formation (see also response to Reviewer 1). Specifically, we quantified the Pearson's correlation coefficients (r) for different VMZ/TJ protein pairs at early (inverted polarity) stages and at the PAP stages (when the junctions surrounding the apical lumen are fully formed). The new data is shown in Figure 4 and Figure S7. We find that at both stages PatJ colocalizes significantly more strongly with Pals1 than with ZO-1 (Fig 4F and 4K). This data is derived from staining endogenous proteins using antibodies as well as from stable cell lines expressing Pals1-GFP and a CRISPR KI cell line expressing mNeon-Green tagged ZO-1. This clearly shows that the VMZ and TJ proteins form separate cortical domains even before the apical lumen is established.

Interestingly, in the inverted stage, Pals1/PatJ and ZO1 localise to cell-cell junctions at the periphery of the lateral interface, facing the ECM (Fig 4E). This is supported by TEM data (Fig 4G). Pals1/PatJ localised closer to the ECM than ZO-1, indicating a clear inversion of polarity in which the VMZ separates the lateral TJ from the ECM-facing apical cortex. Although we cannot fully resolve what happens in between the inverted stage and the AMIS stage, our snapshots of potential intermediate stages (Fig 4H) suggest that VMZ and TJ proteins translocate from the periphery toward the midbody to generate the AMIS (Fig 4I), and while they do so, they switch orientation (i.e. the VMZ proteins move across the TJ proteins to eventually become localised more toward the center). This switch in polarity becomes evident at

the early PAP stages (Fig 4J), in which the VMZ faces toward the apical domain, and ZO-1 faces away from it (toward the newly established lateral domain).

Altogether, our colocalization analysis in 2D and 3D cultures support a model in which TJ and VMZ proteins are spatially separated way before polarity is established, and that the VMZ (i.e. Pals1 and PatJ) act as a spatial (and likely molecular) landmark for VAC docking and fusion at the AMIS.

5. There is a major topological difference between the 2D and the 3D setup and sometimes the authors explanations are too brief to allow the reader to full understand the intricacies.

In the 2D setup, many lateral membrane patches contacting different neighboring cells must somehow become one apical patch with TJ-contacts to all neighbors (topologically a ring) – that also somehow replaces the membrane there. This step is not trivial at all. It 1) either requires "fusion" of lateral lumens of different cell-cell interfaces at lateral 3-vertices. Subsequently, multi-cellular lumens could open towards the top and replace the membrane there. Alternatively, each bicellular lateral lumen could fuse individually with the top "main lumen" generating multiple apical patches that must fuse with each other again to form one apical domain.
Do the authors have more information on how lateral membrane patches fuse to form the large apical "ring", that comprises TJ's contacting all neighbors? We are not recommending generating new data, but simply asking if more information on this is in the current data set. Regardless, some speculation about this would be helpful—this is the last step they depict in Fig 2F.

In contrast, in the 3D setup, there is no lateral lumen. The lumen between the two cells is the future apical side of the cells and does not change position. Instead, new cells that arise by cell division simply get a fraction of this lumen. Is this assumption correct, and if yes could the authors also add a cartoon depicting that, and make the (topological) differences between the 2D and the 3D setup clear in the text? And perhaps a little bit of speculation about whether additional VACs are added after the two cell stage.

Response: Yes, there are indeed major topological differences between the 2D and 3D setup. However, the fundamental mechanism underlying lumen initiation are surprisingly similar. In both systems, VACs transport apical cargo to nascent cell contacts, and in both systems the VMZ and TJ proteins form a junctional ring around the fusion site, which then expands laterally to form a lumen. With regards to the 2D system, we currently don't fully understand how the lateral lumen moves towards the apical side, and how the junctional belt that evidently forms around the lateral lumen is remodelled during this process. We have however observed stages that appear to suggest that the lateral lumen including the junctional belt moves (or is pushed) toward the apex and then spreads across the dorsal part of the cell (see Figure to the right). Resolving this transition and the mechanism underlying this phenomenon is technically challenging as it requires 4D live cell imaging of cells that co-express (at least) a

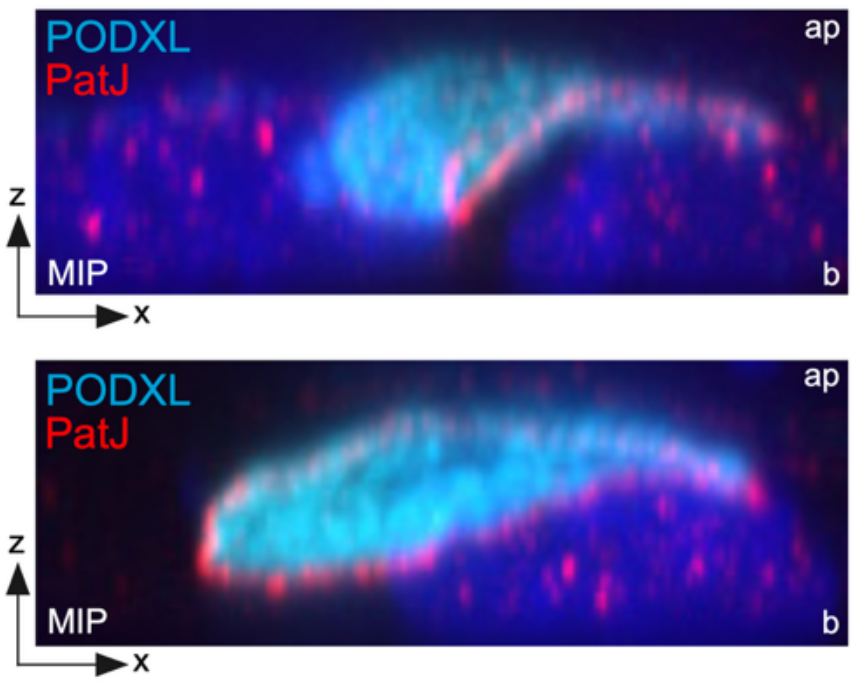

junctional marker (such as ZO1) and an apical marker (such as PODXL). Due to article length restrictions, we have omitted this section - including the potential role of Par proteins in this process - from the discussion.

Our data suggest that VACs are formed primarily in the two-cell AMIS and PAP stages, i.e. when apical proteins need to be redirected from the ECM-facing cortex to the nascent apical lumen. However, in the absence of PatJ, VACs accumulate intracellularly even in multi-cell cysts (see Fig 8C). This suggests that VACs might retrieve mistargeted apical proteins even at later stages of cyst development. Alternatively, VACs that accumulate at the two-cell stages may be inherited to daughter cells as the cyst grows.

6. The last Figure was a bit disconnected with the rest. This could be improved.

Response: We agree with the reviewer and have rearranged the figure panels and the corresponding results section. The SEM data is now shown in Figure 7 and Figure S13. The phalloidin stainings have been moved to the supplements (Fig S12).

In fact, using automated segmentation we have analysed and quantified the organisation and architecture of the marginal zone in WT and PATJ KO cells. This comprehensive analysis revealed marked differences in rod/microvilli density and orientational coherence in PATJ KO cells (Fig 7D-G and Fig S13). This shows that PatJ is an important structural scaffold of the marginal zone, providing mechanistic insight into its function.

Minor points:
Fig 1—it would be helpful to make the panels larger, and perhaps add arrows? Also make it clearer at the start of the paper that you are beginning in 2D and using a calcium switch assay.

Response: We have made these changes and increased the size of the important data panels throughout the manuscript as much as we could.

Fig 1D vesicles on both sides of the plasma membranes?

Response: **Yes, indeed. We have observed "bilateral" fusion events of VACs on either side of the plasma membrane (now shown in Fig 1E). In addition, VACs often dock and fuse right next to each other on the same side of the cell-cell contact interface (e.g. Fig S2C and Fig S4B). We currently have no idea how VAC fusion events are temporally and spatially coordinated. Interestingly, however, in serial section TEM we often see centrioles close to the VAC fusion site (this is now shown in Fig 2C (insets) and Fig S4C and is mentioned in the figure legends). Centrioles were also frequently found in close proximity to intracellular VACs (see Fig S3C). This suggests that the centrosome (or midbody) is not only important for the specification of the AMIS (PMC4976216, PMC7062744) but may also direct VACs to the membrane in the calcium switch assay.**

Fig 1 Captions. D in the last paragraph should be E.

Response: **The figure has changed so this should now be fixed. Thank you for spotting this**

In the text, the authors could add one or two sentences, introducing the calcium switch system, how removing calcium perturbs polarity, and that reintroducing it causes polarity to be re-established allowing to study lumen formation. This will make it easier to understand for non-experts.

Response: **This is now added and a cartoon illustrating the principle of the Ca switch assay is shown in Fig 1A.**

Fig 4b is already the merge.

Response: **Thank you for spotting this – the ZO1 images have been updated. We have changed the colors as well so as to illustrate the spatial separation of TJ and the VMZ more clearly.**

Add the merge in Fig 4C,D.
Response: **This is done. The panel has been updated.**

The interesting membrane staining used in Fig. 6B-E should be briefly described in the main text.

Response: **This is now described briefly in the main text. In addition, we have included in Fig S9A TEM images of cross-sections through 2D MDCK cell monolayers stained with this protocol, which clearly shows strong EM contrast at the lateral but not the apical membrane. In addition, tight junctions appear to exclude the stain and appear electron-lucent, in agreement with our observations in 3D cultures.**

Reviewer #3 (Remarks to the Author):

I co-reviewed this manuscript with one of the reviewers who provided the listed reports. This is part of the Nature Communications initiative to facilitate training in peer review and to provide appropriate recognition for Early Career Researchers who co-review manuscripts.

Reviewer #4 (Remarks to the Author):

The study by Martin et al investigates the processes involved in apical lumen initiation in MDCK cells. Previous studies have yielded quite extensive insight into the molecular players and have suggested models for this process. Briefly, in MDCK cells the cytokinetic remnant (midbody) specifies a site at which the future apical membrane is formed (the apical membrane initation site, or AMIS). Future apical membrane proteins like Ezrin and podocalyxin are then endocytosed from the outer ECM-facing cortex, and transcytosed to the AMIS. This transcytosis was thought to be via standard vesicular transport involving Rab GTPases.

The authors here investigate the different steps of early apical domain formation in MDCK cells using a combination of various ultrastructural approaches and proteomics. They focus their studies on the Crumbs complex (Crumbs–Pals1–Patj). They first use a combination of light microscopy and TEM to investigate the localization of Pals1 at various stages of MDCK polarization. They do this first after a calcium switch in confluent MDCK monolayer, and later in the MDCK 3D cyst culture model. Unexpectedly, they find that Pals1 trafficks to the apical initiation site via large vacuolar apical compartments (VACs) rather than via endocytotic vesicles as previously postulated. Upon fusion of these VACs, Pals1 rapidly accumulates at nascent junction sites. The TEM studies are further elaborated on with FIB-SEM and CLEM microscopy approaches.

To move towards mechanistic insights, the ultrastructural imaging is complemented with APEX2 proximity ligation (with Pals1 as bait) at various time points after calcium switch. This analysis identifies distinct protein profiles at different times, that are consistent with the different steps of trafficking, fusion, and polarity establishment.

Next, the authors investigate the role of Pals1 and Patj in VAC trafficking. As previously reported, loss of either protein results in a multi-lumen phenotype. Imaging at the 2 cell stage indicates this is due to early defects, as knockout cells already show multiple large vacuoles in the doublet stage. The authors furthermore show that inhibiting Rho/ROCK signaling causes similar defects, leading them to postulate that Patj mediates VAC exocytosis through Rho/Rock signaling and actin organization.

All of the imaging and proteomics together convincingly show that VACs are present in MDCK cells in 2D and 3D MDCK cultures, and fuse at cell-cell contacts/AMIS. Timing wise, fusion and junction formation appear to happen close together in time or coincidentally. Together, the data indicate that our current thinking of MDCK polarization, and specifically the transcytosis steps, needs to be refined and incorporate that VACs are a major mode of transport of apical proteins. The data also fit with other papers on pre-assembled apical vesicle structures that are well discussed. The manuscript should therefore be of broad interest to others working on epithelial organization.

Regarding the imaging: I have a question related to the observation of microvilli. Are the authors sure that the structures in the VACs represent microvilli? I ask for two reasons. First, the appearance of these protrusions seems more irregular than I would expect. I do not see actin bundles, and in the supplementary videos the villi often look connected, almost like a network of connected tubules or stacks. Second, in the proximity proteomics, microvillar proteins like EPS8L1, EPS8, BAIAP2, and BAIAP2L1 are detected late, in cluster 3, and not in clusters 1 and 2 during VAC trafficking. Does this not indicate that microvilli are formed only after fusion? After all, the bait protein Pals1 is present on the protrusions during the trafficking phases.

Response: We thank the reviewer for these comments and questions. Yes, we are sure and have now confirmed that the membrane protrusions we observe inside the VACs are microvilli. To support our interpretation, we measured the diameter (width) of the membrane protrusions inside the VACs and compared it to that of apical microvilli in fully mature MDCK monolayers grown on filter inserts (Fig 6D; see also response to reviewer 1). Both apical microvilli and the membrane protrusions inside the VACs had an average diameter of ~110 nm, which matches exactly the expected dimensions of microvilli reported previously by Mooseker and Tilney (1975, PMC2111646) and more recently by others (Ohta et al., 2012, PMID: 22085749). We also measured the diameter of protrusions inside the membrane pits (which we propose are endocytic sites that internalize apical microvilli into VACs) and finger-like protrusions found on the ECM-facing surface. Interestingly, whilst the protrusions inside the membrane pits also had a diameter of ~110 nm, those on the ECM-facing surface were significantly wider (~ 140 nm). This supports our hypothesis that the membrane pits we observe by TEM concentrate and internalize apical cargo and microvilli and that the protrusions outside these pits are distinct from microvilli.

It is true that a central array of actin filaments is a characteristic hallmark of apical microvilli. However, our TEM and FIB-SEM sample preparation protocol (chemical fixation followed by osmification and dehydration on ice) does not preserve actin filaments very well. High-Pressure freezing followed by freeze substitution would be ideal to preserve and visualise the cytoskeleton, which we are not set up for and which is – especially for 3D cultures – not straightforward to do as hydrogels cannot easily be vitrified.

That the microvilli appear connected, stacked or fused in the FIB-SEM volume can be explained by the resolution of this dataset. The voxel size in this dataset is 10x10x30 nm. The axial (z resolution; 30 nm) is determined by the slice pitch, which refers to the volume that is milled away before a new image is acquired. A slice pitch of 30 nm may not always fully resolve individual microvilli along the slicing direction.

That microvillar proteins become enriched with Pals1 at later stages of polarisation is not surprising. In fact, it agrees with the stepwise assembly and maturation of the apical-lateral border. In calcium-depleted cells, Pals1 is expected to be relatively mobile within the VAC microvilli, as it is not anchored to the tight junction scaffold via PatJ (see our work and recent work by the Honigmann lab, PMID: 39112699). At this stage, apical microvillar proteins can be labelled by Pals1, but are not enriched. When VACs are exocytosed at nascent junctions containing TJ proteins, PatJ connects the Crb complex to the TJ scaffold, explaining the increased labelling of TJ proteins at 2h and 4h. As the free apical cortex is formed and the AJC matures, Pals1 and PatJ likely become progressively immobilised at the apical-lateral border. This culminates in the formation of the VMZ, which physically connects TJ and the apical

cortex. Clustering / immobilisation (or perhaps condensation, see PMID: 39112699) of proteins in and around the VMZ is likely to result in more efficient proximity labelling, explaining the late enrichment of microvillar and TJ proteins in the Pals1 proximity proteome. In fact, our serial section TEM data of Pals1 shown in Figure 2E and the SEM data presented in Figure 7 indicate that PatJ plays an important structural role at the marginal zone, being required for the normal assembly and alignment of microvilli-based protrusions that connect adjacent apical cell cortices.

In addition, images in previous publications indeed often are consistent with VACs and as the authors suggest, may simply not have been recognized. Yet there are aspects of previous models of MDCK polarization and transcytosis of apical factors that need to be resolved between prior studies and this manuscript. In particular, work from Bryant and Mostov (2014) suggests that during transcytosis, Ezrin and PODXL do not colocalize. In this manuscript, both are suggested to be present in VACs but a double labeling is not shown. Can the authors resolve whether Ezrin and PODXL traffic together or not?

Response: We thank the reviewer for highlighting this. To ask whether PODXL and ezrin are co-transported to the AMIS in VACs, we did the following experiments:

1. **We performed an antibody uptake assay with labelled PODXL antibody followed by ezrin immunofluorescence (Fig 6E+6F, and S11A+S11B). Because both antibodies are raised in mice, we used an antibody labelling kit to label PODXL with AF647 and ezrin with AF488. A rabbit anti-CGN antibody was used to visualise TJ in the same cells. The data clearly show that internalized PODXL and ezrin efficiently colocalize in VACs and at the AMIS (Fig 6G). Because PODXL antibody was applied to the culture medium, the data also show that VACs originate through internalization from the plasma membrane, and not through, for example, post-Golgi trafficking of newly synthesized proteins.**
2. **We also performed the PODXL antibody uptake assay and ezrin immunostainings in cells stably expressing GFP-Crb3. As expected, internalized PODXL antibody and ezrin colocalized with Crb3 in VACs and at the AMIS (Fig 6G+6H and Fig S11C+D).**

Taken together these new data clearly show that Crb3, PODXL and ezrin are co-internalized into VACs and transported together to the AMIS. This establishes VACs as transcytotic organelles that deliver apical membrane and protein cargo from the ECM-facing plasma membrane to the AMIS.

Regarding the conclusions related to mechanistic insights, I am less convinced by the experiments. The first major conclusion is that Pals1 and Patj loss impairs VAC exocytosis at the AMIS. This conclusion is based on the observation of 'large intracellular vacuoles' and 'ectopic or possibly intracellular lumens' in doublets of knockouts. This could equally well be due to problems with VAC formation or trafficking in the cell, not necessarily with the exocytosis step. Especially since I do not see the suggested accumulation of VACs at the AMIS, but they appear dispersed through the cell.

Response: Since PatJ is present at the AMIS before VACs arrive (Fig 4B), it is likely that PatJ regulates the organisation of the AMIS, rather than the trafficking or assembly of VACs. This is supported by our new observation that TJ proteins are displaced from the AMIS in the absence of PatJ (Fig 8C-8F). A comprehensive quantitative analysis of our SEM data further revealed that PatJ regulates the structure of the marginal zone (Fig 7D-G, Fig S13), which likely plays an

important role in stabilizing the apical-lateral border. Altogether we propose that PatJ connects the TJ to the apical membrane and thereby promotes the incorporation of VACs and junction formation at the AMIS (Fig 9D). In the absence of PatJ, the apical-lateral border is not defined and the spatial landmark for VAC incorporation is missing. This model is supported by our new rescue experiments, which clearly show that restoring the link between TJ and the Crb complex is sufficient to rescue the multi-lumen phenotype of PATJ KO cells.

The second conclusion is that exocytosis defects may be due to defective Rho/ROCK signaling. To support this, they show that inhibiting Rho or ROCK leads to similar defects as knockout of Pals1 or Patj (accumulation of VACs). In addition, in 2D cultures, knockdown of Patj leads to reduced apical F-actin levels. One issue is that the Rho inhibition results appear qualitatively different from the Patj knockout data (compare 7D with 7F): the Rho inhibition vesicles do appear to cluster at the AMIS. But more importantly, the fact that both treatments result in lumen initiation defects does not directly imply that they are functionally related. While Crumbs is indeed involved in Rho/ROCK signaling, the functions of Crumbs are complex and pleotropic. As an apical domain specifying factor and regulator of junctions and actin, the reduced apical actin levels can easily be explained by a lack of Pals1/Patj at the apical membrane domain.

Response: We thank the reviewer for this comment. We have repeated the inhibitor experiments and analysed PAP formation and apical lumen morphology at 24h and 48h in culture (Fig S15). We find that inhibition of Rho (Rhosin), ROCK (Y27632), or myosin-II (Blebbistatin) does not noticeably interfere with PAP formation or single lumen morphogenesis, nor do we observe any consistent defects in the delivery of VACs to the AMIS. In addition, although activation of Rho using CNO3 or inhibition of the exocyst complex using endosidin-2 affected lumen formation and altered lumen shape, these phenotypes were fairly mild and only in part significant (Fig S15E-S15G). These data argue against a major role for actomyosin in lumen initiation and support previous work demonstrating that exocyst is required for the incorporation of apical cargo into the AMIS (Bryant et al, 2010). We also applied these inhibitors to PATJ KO cells, yet neither inhibition nor activation of myosin-II rescued the multi-lumen phenotype or VAC trafficking to the AMIS (see figure on next page). These new observations are now briefly mentioned in the discussion section.

Given the above, we performed a set of additional experiments to understand the underlying cause of the lumen initiation defect in PATJ KO cell (Fig 8C-8G). First, we quantified the VAC accumulation phenotype by measuring the "offset index" (Fig 8G), which clearly confirmed that PatJ is required for the incorporation of VACs into the AMIS. Second, we analysed TJ formation and found that TJ proteins (Cingulin) are severely mislocalised in early and late PATJ KO cysts (Fig 8C-8F). Third, we performed rescue experiments in PATJ KO cells, using various recombinant PATJ constructs (obtained from Alf Honigmann). Somewhat to our surprise, re-expression of full-length PatJ did not rescue the multi-lumen phenotype in our PATJ KO cells (Fig 9B, Fig S14). Interestingly, however, a chimeric construct composed of the PatJ L27 domain fused to ZO-1 fully rescued lumen formation in PATJ KO cells and restored AMIS assembly and the trafficking of VACs to the AMIS (Fig 9A-C). The PatJ-ZO1 chimera also brought Pals1 back to cell junctions, whilst full-length PatJ did not (Fig S14). We conclude that the linkage between the ZO scaffold and the apical Crb complex is required for lumen initiation, and that the lack of this physical tethering rather than deregulation of the actin cytoskeleton is the primary cause of the lumen formation defect in PATJ KO cells. We note that

PatJ re-expression may not be able to restore lumen formation because ZO-1 (the TJ anchor for PatJ) and Pals1 (the apical anchor for PatJ) are markedly downregulated in PATJ KO cells (see also our response to Reviewer 1, question 4).

Manipulation of Rho/actomyosin activity does not restore VAC trafficking and single lumen formation in PatJ KO cells.

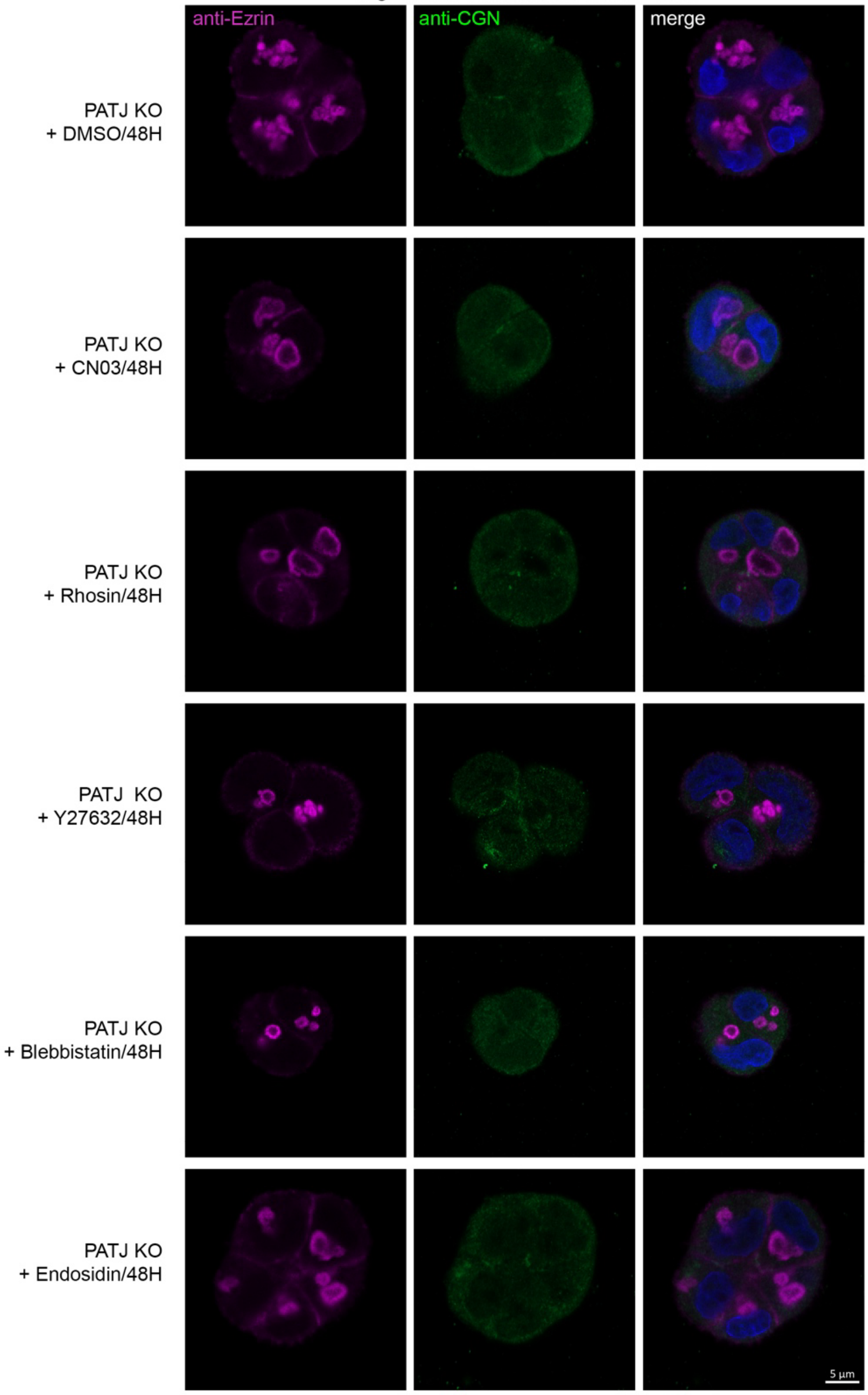

The discussion part of the roles of Pals1 and Patj was also confusing to me. The model presented here is that Pals1 and Patj are at cell-cell contacts, and then VACs deliver apical cargo including Crb3. Assembly of the full Crumbs complex would only take place after VACs fuse at the AMIS. The authors reconcile their finding with previous data concluding that all three Crumbs components traffic via Rab11-positive carriers by stating that the current study is done using antibodies, and previous studies with GFP fusions. However, figures 1 and 2 show that Pals1 is present in VACs. Crb3 and Pals1 would therefore be together in the same compartment. It seems more likely to me that the full Crumbs complex is present in VACs. In addition, for Crb3 the authors rely for their conclusion on the same studies they criticize for using GFP fusions. If the Pals1 and Patj data are not trustworthy, why would the Crb3 data be?

Response: We understand the confusion and should have explained this better. <u>Important to note is that whilst Pals1 and PatJ are internalized into VACs in the calcium switch assay, they are not present in VACs in 3D cultures.</u> This is now clearly supported by our colocalization analyses in 2D and 3D cultures (see Fig 1J+1K and Fig 4F and 4K) and by data presented in Figure 5A-D, all of which is based on antibody stainings against the endogenous proteins. In addition, we have generated a cell line stably expressing GFP-Crb3, which we show is co-internalized with PODXL and ezrin into VACs in the 3D system (Fig 6E-6H).

Our localisation data in 3D cysts do not support the conclusions drawn by Schlueter et al (2010; PMID: 19776356), who based on overexpressed GFP-Crb3, GFP-Pals1, and GFP-PatJ concluded that the entire Crb complex is transported to the AMIS via Rab11-positive carriers (which appear like VACs in most of their images). We believe that much of this discrepancy can be explained by the ectopic recruitment of endogenous proteins to VAC membranes in response to overexpression of one or the other Crb complex protein. For example, we find that even slightly elevated levels of Crb3 can enhance endogenous Pals1 levels on VACs. Taken together, our analyses of endogenous proteins clearly indicate that Pals1/PatJ and Crb3 are initially separated and only "meet each other" after VACs are delivered to the AMIS (see below).

We have amended the paragraph in the discussion accordingly and hope this addresses the reviewers' concerns.

The authors also suggest that Pals1 may act as the VAC cargo receptor by binding to Crb3. Do they mean the receptor that mediates VAC fusion at the AMIS or a receptor that mediates loading of Crb3 into VACs? The first seems unlikely since Crb3 and Pals1 are both in VACs. The second is possible but would have no bearing on the delivery of VACs.

Response: We apologise for this misunderstanding and agree we should have explained this better. <u>We point out again that there is an important difference between VACs in the Ca-switch experiment and those in 3D cultures. In 2D, both Pals1 and PatJ are internalized into VACs together with Crb3 (Fig 1E and Fig S2, and see Figure below). By contrast, in 3D cysts, Pals1 and PatJ follow TJ proteins and accumulate at the AMIS before VACs arrive (Fig 4 and 5A-D), whereas GFP-Crb3 is localised at the ECM-facing cortex and internalized together with PODXL and ezrin into VACs, which are then transported to the AMIS (Fig 6G+6H and Fig S11).</u>

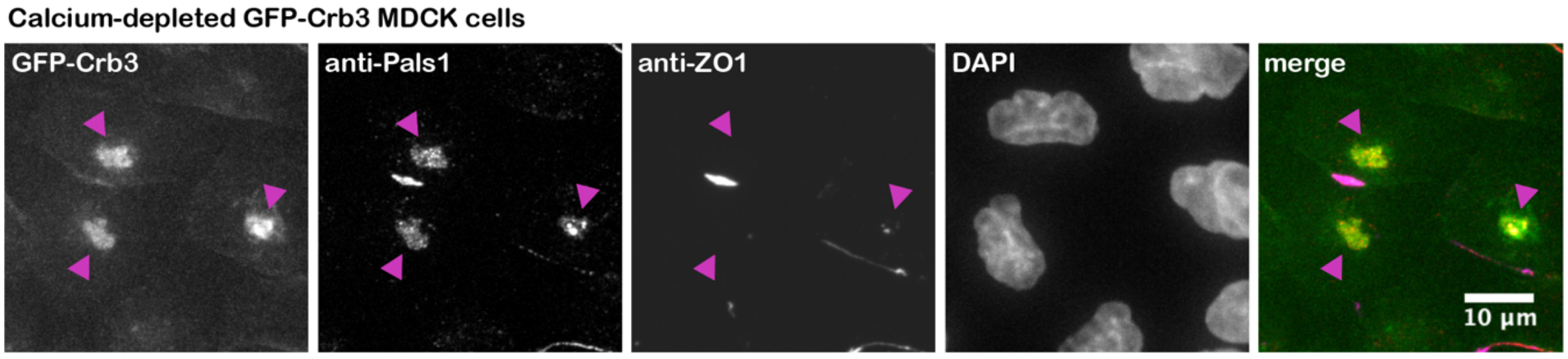

Our KO data indicate that PatJ and Pals1 are required for the docking, exocytosis, and/or fusion of VACs at the AMIS. Because Pals1 interacts directly with the Crb3 C-terminal ERLI motif, we speculated that Pals1 may act as an "AMIS-localised VAC receptor". This model also implies a direct role for Crb3 as a "VAC-localised AMIS targeting signal". Although this is in line with data showing that downregulation of Crb3 perturbs the trafficking of PODXL to the AMIS (Schlueter et al., 2010; PMID: 19776356), other work by Keith Mostov and colleagues demonstrated that loss of PODXL also prevents the targeting of apical proteins to the AMIS, including Crb3 and Syntaxin3 (Bryant et al., 2014; PMID: 25307480). This might suggest redundancy or, alternatively, that PODXL and Crb3 cooperate in delivering VACs to the AMIS. Because of manuscript length restrictions and uncertainties regarding the underlying mechanisms of VAC transport and exocytosis we have omitted this discussion from the manuscript.

Before publishing this work, it will be necessary to either perform additional work to strengthen the mechanistic insights, or to significantly tone down the mechanistic conclusions. In either case, the confusing contradiction in the discussion should be resolved. To strengthen the mechanistic insights, the authors should show that loss of Pals1 specifically affects exocytosis by performing time-lapse imaging of VACs and demonstrating that their trafficking to the AMIS is not affected. In addition, the authors should show that composition and structure of VACs is not affected by Pals1 loss. That the defects in Rho signaling and Crumbs complex loss are functionally related also needs to be substantiated. For example, can the Pals1 defects be restored by increasing Rho activity?

Response: We have significantly strengthened the mechanistic insight into the role of PatJ in lumen initiation. (A) We have quantified the lumen initiation defect in PATJ KO cells, demonstrating that PatJ is critical for the incorporation of VACs into the AMIS. (B) We have quantified the architectural defects in the marginal zone (MZ) of PATJ KO cells, showing that PatJ is critical for its normal assembly. (C) We have performed rescue experiments in PATJ KO cells, demonstrating that the TJ-VMZ linkage is critical for lumen formation. Altogether the data indicate that PATJ acts as an important structural and functional scaffold of the MZ and connects the TJ to the apical cortex. In its absence, the MZ is not properly formed and TJ and AMIS assembly is perturbed, leading to the loss of a spatial signal for VAC exocytosis and their subsequent accumulation in the cytoplasm. The discussion section was altered accordingly.

We further show that Ezrin still localises to VACs in the absence of PATJ (Fig 8C+E) and that these VACs contain internalized PODXL (Fig 8D+F), indicating that VAC composition is not notably altered in PATJ KO cells. Moreover, based on our re-evaluation of Rho signaling inhibitors in WT and PatK KO cells, we currently have no evidence for a direct role of actomyosin dynamics in lumen initiation (see Fig S15 and corresponding discussion section).

Unfortunately, time-lapse imaging of early-stage 3D cysts turned out to be challenging. In our hands, MDCK cells embedded in Matrigel undergo cell cycle arrest and/or apoptosis when challenged by live cell microscopy. All our attempts to minimize photodamage and toxicity (using wide-field microscopy instead of confocal illumination, reducing laser power/exposure times etc) or improve viability (culture medium with or without phenol red etc) failed.
