# Supplementary material for: Bulk delivery of a preassembled apical surface initiates epithelial lumen formation
Source: Nat Commun. 2026 Aug 7;17:9520. doi: 10.1038/s41467-026-75503-w (PMC13547361; doi:10.1038/s41467-026-75503-w)

Figure 7A: Characterisation of PatJ KO cells

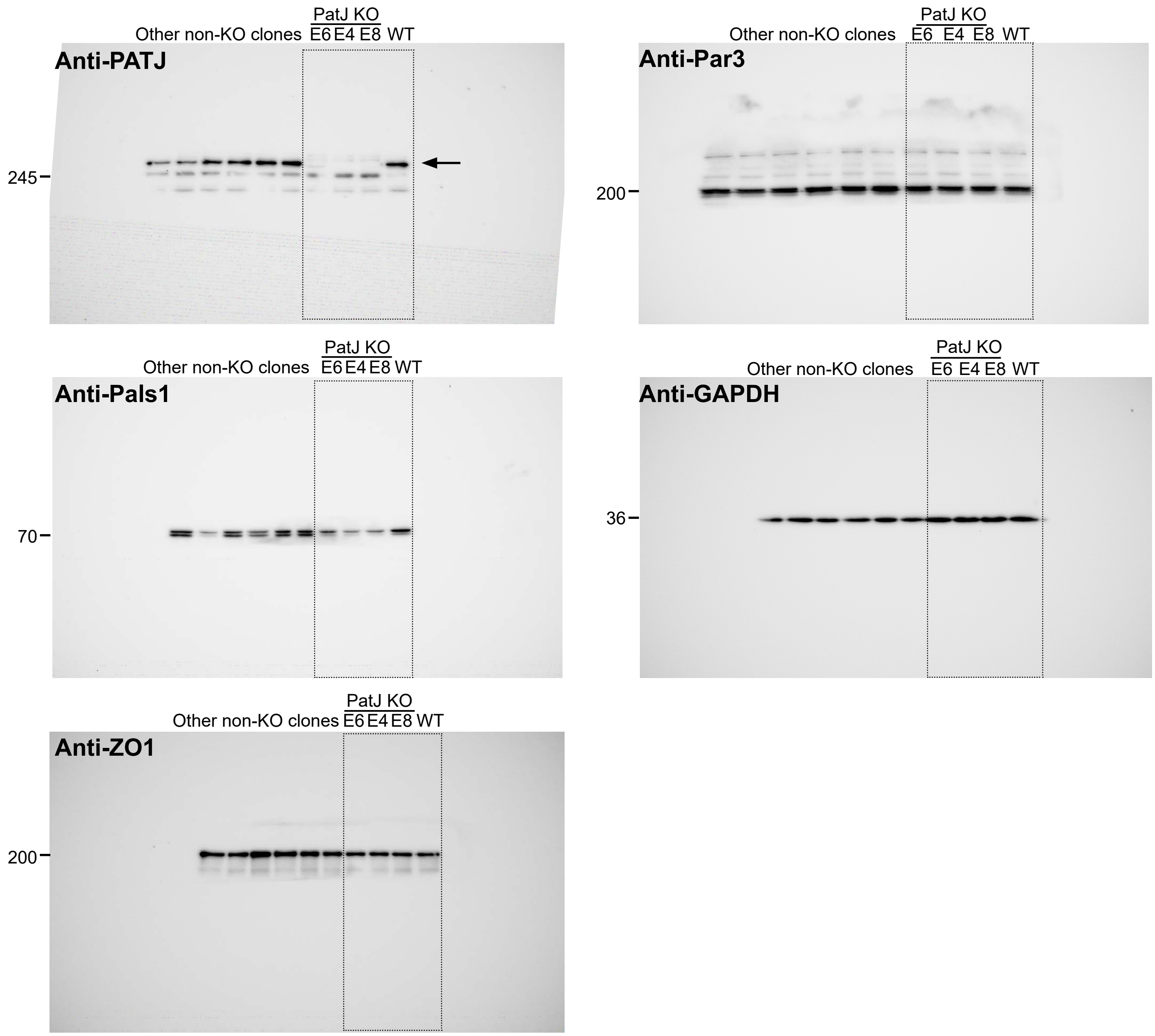

Figure 7A: Quantification of Pals1 levels in PatJ KO cells

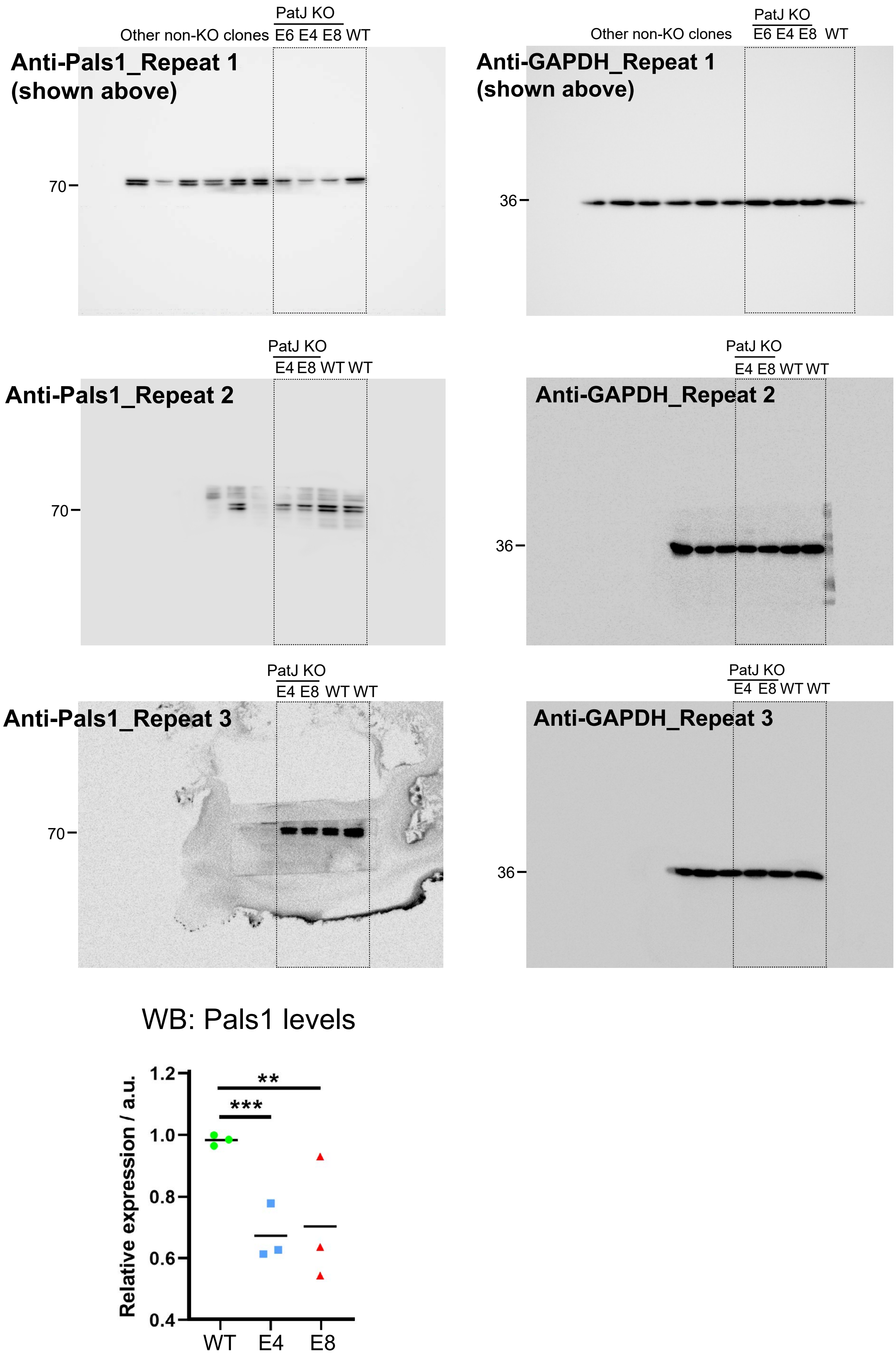

Supplement: Supplementary file 5 [file 41467_2026_75503_MOESM10_ESM.zip › Figure 7A_WB original blots.pdf]
